# Combined Liver-Kidney Transplantation for Hepatorenal Syndrome

**Published:** 2015-08-01

**Authors:** V. Kanubhai Sutariya, A. Tank, P. Ramanlal Modi

**Affiliations:** Department of Gastrointestinal Surgery and Liver Transplantation, IKDRC-ITS, Civil Hospital Campus, India

**Keywords:** Combined liver-kidney transplantation, Hepatorenal syndrome, Renal replacement therapy

## Abstract

Among various complications of end-stage liver disease, hepatorenal syndrome has the highest mortality. Patients with both end-stage liver disease and end-stage renal disease are candidates for combined liver-kidney transplantation. However, patients with cirrhosis with decompensation presenting in the form of hepatorenal syndrome, are also likely candidates for the procedure. Herein, we present a patient who underwent combined liver-kidney transplantation for hepatorenal syndrome.

## INTRODUCTION

Patients with liver cirrhosis develop decompensation in various forms like hematemesis, intractable ascites, hepatocellular carcinoma, hepatic encephalopathy, and hepatorenal syndrome (HRS). Among these complications, type 1 HRS carries the worst prognosis. Type 1 HRS is rapidly progressive renal dysfunction while type 2 HRS is slowly developing renal dysfunction in patients with liver cirrhosis. Various reports indicate that the median survival of patients with type 1 HRS is less than two weeks [1, 2]. With an improved understanding of the pathophysiology and underlying mechanisms leading to hepatorenal syndrome, new drug therapies have been introduced during past two decades. Recently, a meta-analysis of various randomized controlled trials evaluating effects of drugs like terlipressin has been published [3]. Despite successful drug treatment approaches, to date, the only definitive treatment for type 1 HRS is either liver transplantation or combined liver-kidney transplantation (CLKT). The decision to perform CLKT is straightforward for patients with end-stage liver and renal disease and for patients with severe chronic renal failure. However, it is less clear for potentially reversible causes of renal failure like hepatorenal syndrome. Hereon, we present a patient with cirrhosis of liver complicated by HRS who underwent CLKT.

## CASE REPORT

A 42-year-old male patient presented with complaints of distension of abdomen, decreased urine output and edema of feet. He had a history of melena, paracentesis and upper GI endoscopy with banding of grade 3 varices. He had also history of type 2 diabetes mellitus. He was not alcoholic and his autoimmune antibody profile was negative. Twenty-four-hour urinary copper and serum ceruloplasmin were normal. Kayser Fleischer’s ring was not found on slit lamp examination. His lab findings on presentation included a total billirubin of 1.6 mg/dL (direct 1.0 mg/dL, indirect 0.6 mg/dL), alanine aminotransferase of 55 U/L, aspartate aminotransferase of 35 U/L, alkaline phosphatase of 120 U/L, international normalized ratio (INR) of 1.49, serum albumin of 2.8 g/dL, and a serum creatinine of 1.62 mg/dL. His Child-Turcotte-Pugh score was 8 and Modified End-stage Liver Disease (MELD) score was 11. Once diagnosed having decompensated cryptogenic cirrhosis of liver, he was placed on liver transplant waiting list. He was on diuretic on presentation. He was initially managed with plasma expander and omission of diuretics. Creatinine rose above 2 mg/dL. His urinalysis showed no evidence of microscopic proteinuria or microalbuminuria. All other possible causes of renal failure were ruled out. He was thus diagnosed with HRS type 1. The patient did not respond to combination therapy with albumin and terlipressin and his serum creatinine increased to 5 mg/dL. He was placed on hemodialysis. The patient was kept on hemodialysis for 10 weeks when he received a liver from a cadaver donor. Donor was a 50-year-old brain-dead woman who died of MVA. At that time, considering patient’s renal dysfunction, CLKT was carried out. The patient was maintained on continuous renal replacement therapy during perioperative period. The inferior epigastric artery was preserved to avoid wound-related complications. Postoperatively, the patient did not require any kinds of renal support. After two years of follow-up, the patient was maintained well on tacrolimus, mycophenolate mofetil and steroid.

## DISCUSSION

Renal failure occurs in up to 10% of patients with advanced liver disease and even more frequently in patients on waiting list. Renal dysfunction in hepatorenal syndrome is mostly reversible, as indicated by a report of successful transplantation of kidneys from dying patients with hepatorenal syndrome to patients without liver failure. HRS can only be diagnosed after all other causes of renal failure have been excluded; the possible causes include obstruction, volume depletion, and acute tubular necrosis. All diuretics should be stopped and fluid challenge with isotonic saline should be administered to exclude volume depletion. The most probable pathogenesis of HRS is hypoperfusion of kidneys resulting from combined effects of intrarenal arteriolar vasoconstriction and peripheral vasodilatation mainly in splanchnic circulation. 

Because of potential reversibility with liver transplantation (LT) alone, HRS is not being considered routinely for CLKT [4]. However, patients with HRS may develop end-stage renal disease after LT alone. The longer waiting time for LT, in recent times, has led to a rise in the incidence of pretransplantation renal dysfunction. In such circumstances, prolonged HRS and long-term renal replacement therapy (RRT) can lead to permanent renal damage so that the renal function may not be adequate after LT alone [5]. In addition, no studies are available reporting renal recovery and long-term renal function after prolonged RRT. This uncertainty, along with known nephrotoxicity of calcineurin inhibitors, has lead to a trend to performing CLKT when renal recovery is not possible. However, the duration of HRS and RRT beyond which the outcome of CLKT is better than that seen with isolated LT is not specified, yet. Therefore, better predictors are required to select patients with permanent renal injury. At the present time, in absence of solid data for prediction of renal recovery, it is justified to perform CLKT in patients with HRS who have been on RRT for more than eight weeks. This may be shortened to six weeks in patients with previous episodes of acute renal failure [6, 7].
